# Social Media Landscape of the Tertiary Referral Hospitals in China: Observational Descriptive Study

**DOI:** 10.2196/jmir.9607

**Published:** 2018-08-09

**Authors:** Wei Zhang, Zhaohua Deng, Richard Evans, Fei Xiang, Qing Ye, Runxi Zeng

**Affiliations:** ^1^ Institute of Smart Health School of Medicine and Health Management Huazhong University of Science and Technology Wuhan China; ^2^ Business Information Management and Operations University of Westminster London United Kingdom; ^3^ Tongji Hospital Tongji Medical College Huazhong University of Science and Technology Wuhan China; ^4^ School of Journalism and Communication Chongqing University Chongqing China

**Keywords:** social media usage, best tertiary hospitals, China, Sina Weibo, WeChat

## Abstract

**Background:**

Social media has penetrated all walks of life. Chinese health care institutions are increasingly utilizing social media to connect with their patients for better health service delivery. Current research has focused heavily on the use of social media in developed countries, with few studies exploring its usage in the context of developing countries, such as China. Tertiary hospitals in China are usually located in city centers, and they serve as medical hubs for multiple regions, with comprehensive and specialized medical care being provided. These hospitals are assumed to be the pioneers in creating official social media accounts to connect with their patients due to the fact that they appear to have more resources to support this innovative approach to communication and health care education.

**Objective:**

The objective of our study was to examine China’s best tertiary hospitals, as recognized by The National Health Commission of the People’s Republic of China (NHCPRC), and to map out the landscape of current social media usage by hospitals when engaging with patients.

**Methods:**

We examined the best 705 tertiary hospitals in China by collecting and analyzing data regarding their usage of popular Chinese social media apps Sina Weibo and WeChat. The specific data included (1) hospital characteristics (ie, time since established, number of beds, hospital type, and regions or localities) and (2) status of social media usage regarding two of the most popular local social media platforms in China (ie, time of initiation, number of followers, and number of tweets or posts). We further used a logistic regression model to test the association between hospital characteristics and social media adoption.

**Results:**

Of all, 76.2% (537/705) tertiary referral hospitals have created official accounts on either Sina Weibo or WeChat, with the latter being more popular among the two. In addition, our study suggests that larger and newer hospitals with greater resources are more likely to adopt social media, while hospital type and affiliation with universities are not significant predictors of social media adoption among hospitals.

**Conclusions:**

Our study demonstrated that hospitals are more inclined to use WeChat. The move by hospitals from Sina Weibo to WeChat indicates that patients are not satisfied by mere communication and that they now place more value on health service delivery. Meanwhile, utilizing social media requires comprehensive thinking from the hospital side. Once adopted, hospitals are encouraged to implement specific rules regarding social media usage. In the future, a long journey still lies ahead for hospitals in terms of operating their official social media accounts.

## Introduction

Social media is now an indispensable part of human life. The number of globally active social media users reached a new high of 2.8 billion in 2017, representing 37% worldwide penetration. Facebook has the highest number of active users (1871 million), followed by Facebook Messenger, WhatsApp, and YouTube [1]. The ease of use of current social media websites and apps, including their uncomplicated operation and simplicity in generating user content and instant messages, allows people to connect in a more convenient manner across space and over time. It is undeniable that social media has enabled the reshaping of how we identify, get to know, and maintain relationships with others.

In recent years, health care professionals and institutes have begun to realize the benefits of using social media for building patient-physician relationship and for health care service delivery [2,3]. Evidence suggests that social media usage by hospitals and health care professionals contributes to the increase in hospital website visitors, brand establishments, and patient recruitment for research projects [4-6]. In the United States, 70% of hospitals use Facebook [7] while most hospitals utilize at least one social media platform [8]. Wong et al [9] have investigated the adoption of social media by children’s hospitals in the United States and concluded that social media can serve as a channel for providing health care education and community engagement. In Western Europe, hospitals in the Netherlands and the United Kingdom are seen as pioneers in health care social media adoption, and the most widely adopted social media platforms by hospitals are YouTube, LinkedIn, and Facebook [10]. Studies have also discovered certain patterns of social media usage among hospitals; for example, large, urban, not-for-profit hospitals and hospitals that are affiliated with universities or health systems are more likely to operate official social media accounts [7,8]. Despite the recognized rise in social media usage by hospitals, a survey conducted in 2014, which explored the official websites of Italian local health authorities and public hospitals, found a low social media usage [11]. Researchers [12-14] have also established that the effects of social media on patient engagement vary between hospitals and that substantial differences exist between social media adoption and its effects. Further content analysis has revealed that the current social media usage in public health agencies has enabled the centralization of information distribution, rather than interactive communication, and a strategic communication plan has been deemed necessary for expanding the reach of social media and fostering interactivity and engagement among patients [12]. A study on local health care departments in the United States also indicated that although Twitter has been widely adopted, its primary use has been a one-way communication by hospitals on personal health care topics and organization-related information [13].

All these studies have contributed to an understanding of how hospitals use social media and its impact. However, almost all research conducted has been in the context of developed countries, with less attention being paid to developing countries, such as China. Due to differences in health care systems and local culture, social media usage by hospitals in developing countries may vary greatly from similar hospitals in developed countries.

Over the past several years, social media has been gradually adopted by China’s hospitals for various reasons, such as building harmonious physician-patient relationships, reducing a hospital’s burden on health service delivery, and improving public health literacy. In China, social media usage offers at least the following two benefits to the public: (1) It is a reliable source of health information. Enabled by information communication technology, everyone effortlessly becomes a self-publisher. Web-based health information can be provided by anyone who has access to the internet, although the trustworthiness of the information is not always guaranteed. With the increase in social media accounts operated by recognized health institutions, the public can now increasingly trust health information posted online and not be misled by unrecognized self-publishers. (2) It is a convenient and precise match for medical consultations in hospitals. For patients paying a first-time visit to a hospital, the interaction with the hospital’s staff via social media is of great help in finding the right physician within a short period of time. Otherwise, patients may waste lots of time in, for example, figuring out the right department and the appropriate physician who specializes in their illness.

**Figure 1:**
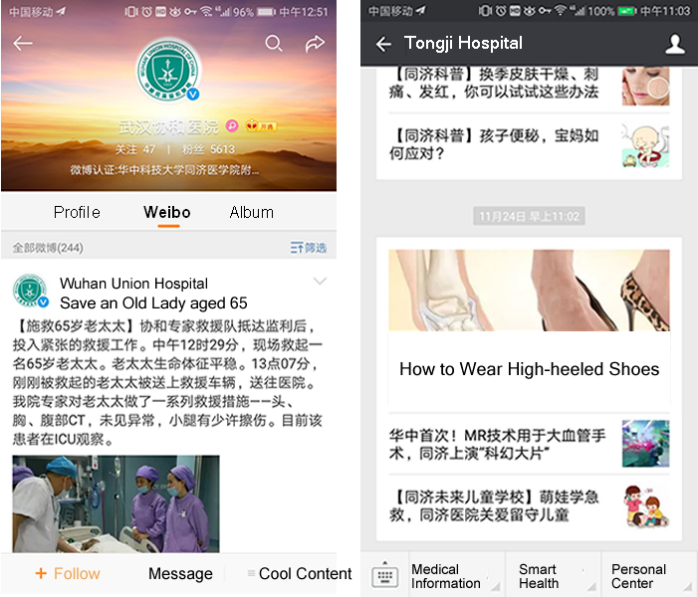
Social media usage by two hospitals (Sina Weibo on the left and WeChat on the right).

This study aimed to provide an empirical analysis of social media usage by the best hospitals in China, one of the largest developing countries. Although China has 29,719 hospitals, which were being used by more than 1.3 billion individuals by the end of July 2017 [15], the vast majority of individuals in China want to go to the best hospitals and spare no efforts when seeing a doctor; this is deemed to increase the pressure on China’s health care system, especially on the best tertiary hospitals in the country. As a result, the best tertiary hospitals are typically overcrowded and have a strong desire to improve their patient-centered communication and health care services. Meanwhile, 57% of individuals in China are active on social media, spending an average of 1 hour and 50 minutes on social media platforms per day [1]. The most popular social media platforms in China are those tailored toward Chinese nationals, which include WeChat (67%), Youku, Sina Weibo (45%), and Tencent Weibo (31%); all of these platforms are different from Facebook and Twitter in terms of their functionality and bespoke features for user cultures. For example, WeChat is a closed social networking platform that allows users to connect with everyone in their circle, whereas strangers and the general public are not allowed to review or comment on their personal pages. In addition, WeChat embraces instant messaging with text, image, voice, and video chat; these features make the social media usage by China’s best hospitals unique [16]. Hospitals implement WeChat to provide services to patients, including setting up appointments with physicians and providing health education in general, while their usage of Sina Weibo focuses on instant communication with patients and health education. Figure 1 presents an example of WeChat and Sina Weibo adoption by 2 hospitals in China.

The following research questions (RQs) were answered in this paper:

RQ 1: How has social media been adopted and utilized by China’s best tertiary hospitals over time?

RQ 2: What factors are associated with the impact of social media on China’s best tertiary hospitals?

## Methods

### Study Sample

In total, 705 of China’s best tertiary hospitals, as recognized by the NHCPRC, were selected for this study. These hospitals were assumed to be the pioneers in creating official social media accounts to connect with their patients due to the fact that they appear to have more resources to support this innovative approach to communication and health care education. In China, hospitals are classified into three categories according to their ability to provide medical care and education and to conduct medical research: (1) primary, (2) secondary, and (3) tertiary. For example, tertiary hospitals are usually located in city centers, and they serve as medical hubs for multiple regions, with comprehensive and specialized medical care being provided [17]. Furthermore, each hospital in China is graded as either A, B, or C based on an overall evaluation of medical technology adoption, medical equipment, patient safety, hospital management, etc. Grade “A” indicates the best quality, whereas grades “B” and “C” indicate lower qualities. In our study, the best tertiary hospitals are defined as those categorized as tertiary and graded A. We followed the most recent updates on the NHCPRC’s official website using the correct data as of June 1, 2017. The sample of 705 hospitals is spread across 31 provincial governments in Mainland China, and the number of hospitals in each provincial government ranges from 1 to 66.

### Data Collection

Data collection consisted of two parts: (1) collection of hospital attributes and (2) collection of data on social media usage. For part 1, we collected data pertinent to the characteristics of the hospitals, including number of beds, geographical location, year of establishment, and hospital type (comprehensive or specialized hospitals), from their official websites. For part 2, we focused on the hospitals’ official presence on Sina Weibo and WeChat, the most popular social media platforms in China. Specifically, the following data were included: date of account creation, number of followers, number of tweets or posts, the most recent update time, and the major functionality offered on their social media account.

To ensure the comprehensiveness of data on official social media usage, we explored the required information using two approaches [7]: (1) searching the hospitals’ official websites to check for social media accounts and (2) searching verified account information on Sina Weibo and WeChat using various keywords, including full name and abbreviation(s). We developed an initial data collection framework with detailed instructions. To collect data, three research assistants were employed and trained for the work. To ensure that the social media accounts were measured consistently, we removed the verified accounts of the separate departments or sections of the same hospital, for example, Department of Medicine of Hospital A or Nursing Station of Hospital B; this was done because these verified accounts only partially represented each hospital and not the hospital as a whole.

In addition, we further assessed the top 10 most popular hospitals on Sina Weibo as a case study for the availability of their data to explore the contents initiated by these hospitals on their social media accounts and their interaction with the general public.

### Data Analysis

To analyze the collected data, a unique coding scheme was developed. Specifically, the coding consisted of two parts: the first was for hospital characteristics and the second for hospital social media account. In the first part, the Year of Establishment included 4 codes (1=before 1900, 2=1900-1949, 3=1950-1999, and 4=2000 onwards); Number of Beds included 5 codes (1=below 500, 2=501-1000, 3=1001-1500, 4=1501-2000, and 5=over 2000); and Status of Affiliation included 2 codes (0=no affiliation with a university and 1=affiliation with a university). Furthermore, we divided Mainland China into three regions according to the National Bureau of Statistics of China. The three regions were coded as Eastern, Central, and Western, with Eastern having the highest economic development level and Western having the lowest economic development level. In addition, hospital types were divided into “specialized” and “comprehensive.” Regarding social media usage, our assessment was based on whether the hospital had an active account on Sina Weibo or WeChat. Meanwhile, Year of Verification, Number of Follows, and Number of Tweets or Posts were also coded.

To answer RQ1, we mapped out social media usage among the 705 hospitals across time using ArcGIS. To answer RQ2, we used the logistic regression model to analyze the correlation between hospital characteristics and their adoption of social media platforms.

## Results

### Descriptive Study

**Figure 2:**
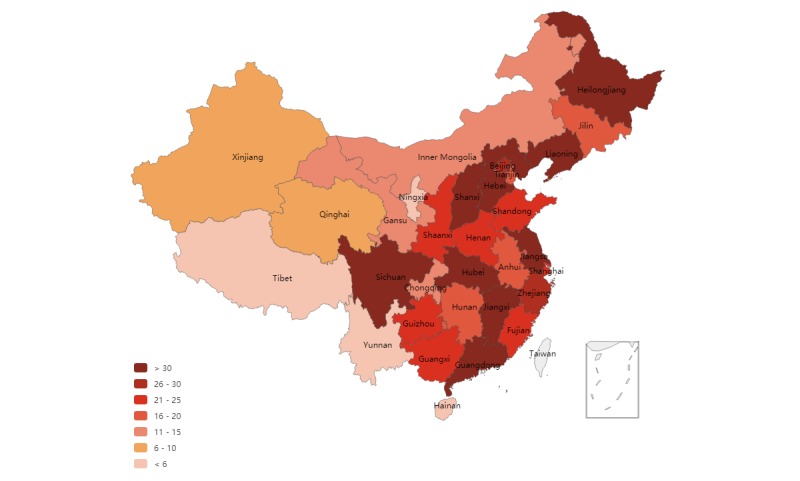
Distribution of the best tertiary hospitals across Mainland China.

**Figure 3:**
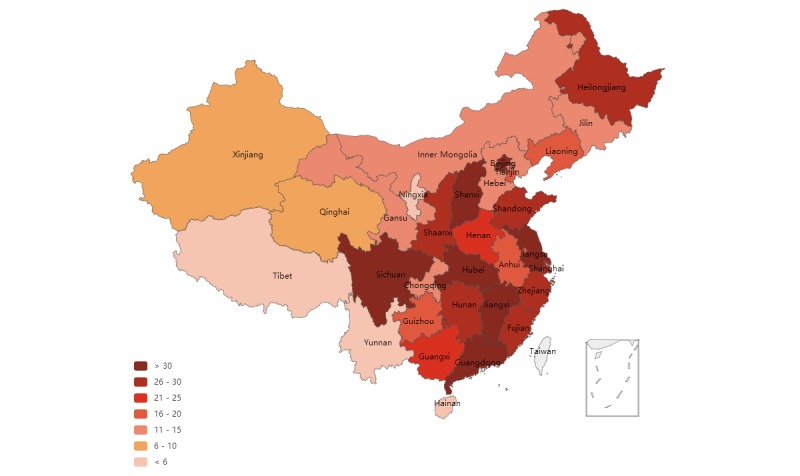
Diffusion of Sina Weibo and WeChat among the best tertiary hospitals in Mainland China.

The distribution of the studied 705 hospitals is presented in Figure 2. The best medical resource in Mainland China is unevenly distributed across the three regions. It is evident that the Eastern region has more of the best tertiary hospitals than the Central and Western regions. Among these hospitals, 76.2% (537/705) have official accounts on either Sina Weibo or WeChat (Figure 3).

#### Social Media Adoption Is Prevalent Among the Best Tertiary Hospitals in China, With WeChat Being More Popular Than Sina Weibo

Among the 537 hospitals that have opened at least one official social media account, 267 use Sina Weibo and 446 use WeChat. Table 1 provides the number of social media accounts by province. In total, 176 hospitals have opened accounts on both Sina Weibo and WeChat. The use of Sina Weibo greatly varies across the three regions. Over half of the accounts (142/267, 53.2%) were identified in the Eastern region, whereas only 20.9% (56/267) were found in the Central region and 25.8% (69/267) in the Western region. Meanwhile, the earliest hospitals using Sina Weibo appear in the Eastern region, in Beijing and Tianjin in around 2009. Regarding the use of WeChat, the diffusion of hospitals is distributed evenly across the three regions. Specifically, the Eastern and Central regions have 182 and 173 hospitals using WeChat, respectively. The earliest WeChat accounts were identified in 2014, with 20 hospitals from 7 provinces across the three regions being identified. Surprisingly, Heilongjiang, located in the Central region of China, outnumbered other provinces with its 12 WeChat accounts. We also found that the total number of best hospitals that adopted WeChat outnumbered those that adopted Sina Weibo. In total, 23 out of 31 provinces had more hospital WeChat accounts than Sina Weibo accounts, excluding 3 provinces from the Eastern (Beijing, Shanghai, and Shandong) and 4 provinces from the Western (Chongqing, Inner Mongolia, Yunnan, and Ningxia) regions.

#### Social Media Penetration Rates Vary Across Provinces, With the Best Tertiary Hospitals From the Central and Western Regions Catching Up

The best tertiary hospitals from almost all provinces showed interest in adopting Sina Weibo or WeChat. The best hospitals from 29 of the 31 provinces have opened their account on Sina Weibo, except for Hainan from the Eastern Region and Tibet from the Western region. All of the best hospitals in Beijing and Ningxia Hui autonomous region have opened accounts on Sina Weibo, while Hebei, in the Central region, has the lowest penetration rate on Sina Weibo (6%). Regarding the total number of official accounts, Guangdong and Beijing have the highest numbers with 30 accounts, followed by Shanghai (17 accounts). Regarding WeChat, 30 out of the 31 provinces have at least one best hospital with a WeChat account, except for the best hospitals in Tibet. Hubei province has the highest penetration rate at 97.2%. On the other hand, Yunnan has the lowest penetration rate at 20%. Regarding the total number of official accounts on WeChat, Guangdong has the highest number (41), followed by Hubei (35).

**Table 1:** Sina Weibo and WeChat diffusion among the 705 hospitals in Mainland China.

Province		No. of Best Hospitals			No. of Sina Weibo and WeChat accounts		No. of Sina Weibo accounts		No. of WeChat accounts
**Eastern Region**									
	Beijing	30			45		30		15
	Tianjin	17			18		8		10
	Hebei	32			13		2		11
	Liaoning	36			17		5		12
	Shanghai	24			28		17		11
	Jiangsu	38			36		15		21
	Zhejiang	26			29		13		16
	Shandong	21			27		14		13
	Guangdong	66			71		30		41
	Fujian	24			29		8		21
	Hainan	5			3		0		3
**Central Region**									
	Hubei	36			47		12		35
	Hunan	20			28		10		18
	Henan	24			24		7		17
	Anhui	20			18		2		16
	Jiangxi	33			34		8		26
	Shanxi	32			31		8		23
	Jilin	20			14		3		11
	Heilongjiang	31			30		5		25
**Western Region**									
	Guangxi	25			23		8		15
	Chongqing	11			14		9		5
	Sichuan	36			43		16		27
	Guizhou	23			16		5		11
	Inner Mongolia	13			11		9		2
	Yunnan	5			3		2		1
	Tibet	1			0		0		0
	Shaanxi	25			28		11		17
	Gansu	12			15		5		10
	Qinghai	8			6		1		5
	Ningxia	3			4		3		1
	Xinjiang	8			8		1		7
Total			705	713		267		446	

**Figure 4:**
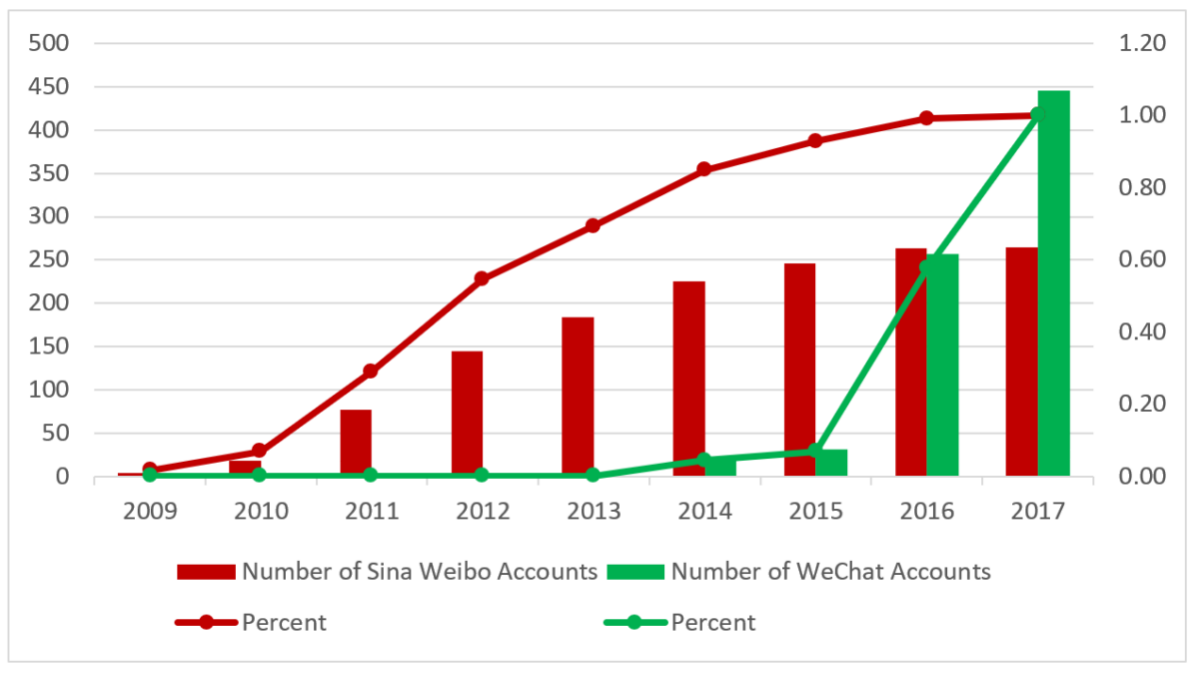
Social media usage among the best tertiary hospitals over time (2009-2017).

#### New Adopters Have Emerged, With Inactive Members Also Appearing

**Figure 5:**
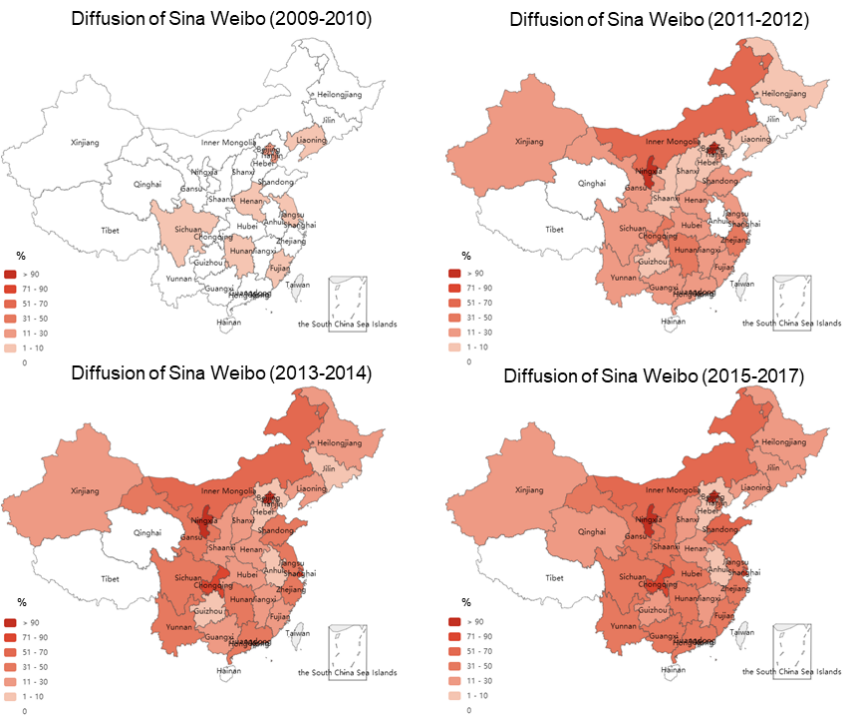
Diffusion of Sina Weibo across the best tertiary hospitals over time (2009-2017).

Over the past decade, the social media landscape of China’s best hospitals has dramatically changed (Figure 4). Regarding Sina Weibo, the best hospitals have already started to explore this innovative communication tool since its establishment in 2009. Adoption peaked in 2012, with 68 best hospitals using the platform; after that, new adopters began to decline. Two best hospitals, one from Shandong and the other from Anhui province, have recently adopted Sina Weibo in the first half of 2017. Figure 5 provides the diffusion of Sina Weibo use across provinces from 2009 until mid-2017. Meanwhile, we also found that 25.5% (68/267) hospitals across 24 provinces had inactive Sina Weibo accounts during the past 6 months.

**Figure 6:**
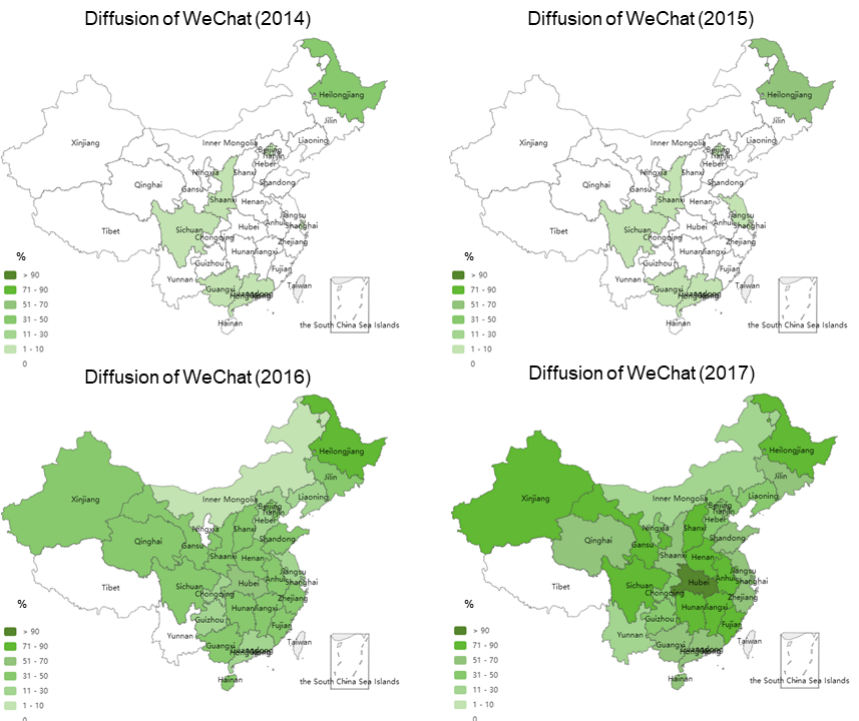
Diffusion of WeChat across the best tertiary hospitals over time (2009-2017).

We identified a similar diffusion trend for WeChat. However, WeChat has fewer inactive hospital accounts. WeChat introduced an account verification service in the late 2012, with the first verified account among the best hospitals appearing in 2014. In total, 20 early WeChat adopters covered three regions, including 12 adopters from Heilongjiang, 3 from Beijing, and the remaining from Shanghai, Guangdong, Guangxi, Sichuan, and Shaanxi. In 2015, 11 hospitals joined WeChat, while in 2016, 227 did so. During the first half of 2017, 197 new best hospitals started using WeChat to connect with the public, indicating large-scale and continuous adoption. Figure 6 shows the diffusion of WeChat across provinces from 2009 until mid-2017. However, we also noted that WeChat accounts of 6.5% (29/446) best hospitals across 13 provinces had been inactive for the past 6 months.

### Social Media Adoption and Hospital Characteristics

To examine the association between social media adoption and hospital characteristics, we performed logistic regression analysis. We found that Sina Weibo and WeChat adoptions were differently predicted using hospital characteristics. Regarding Sina Weibo, a hospital’s year of establishment (*P*=.09), hospital’s affiliation with a university (*P*=.07), and hospital type (*P*=.007) were significant, whereas, regarding WeChat, number of beds (*P*=.003) and regions (*P*=.02) were significant. We used Sina Weibo as an example to unravel the popularity of social media in the best hospitals, exploring visibility for their followers. Of these, the average number of followers was 37,512.26 (min 12, max 1,576,347, SD 151,038.36), whereas the average number of tweets posted was 1446.16 (min 0, max 30,047, SD 279,674). We further treated the number of tweets posted as a dependent variable, the number of followers as an independent variable, and hospital characteristics as control variables. The linear regression indicated a strong correlation between the number of tweets and the number of followers (*F*_1_=38.21; *P*<.001). The list of the top 10 most popular verified hospital accounts on Sina Weibo is presented in Table 2. Among the top 10 hospital accounts, 9 are from the Eastern region of China, with 1 being from the Western region. In the Eastern region, Beijing, China’s capital city, has 7 such hospital accounts. Meanwhile, 7 of the 10 were specialized hospitals, with children’s hospitals receiving greater attention from the public on social media.

We further explored the content of the social media posts and the interactions through these by collecting the top 10 commented tweets or blogs from each of the 10 hospitals. Generally, the 100 posts can be divided into 5 categories on the basis of their content: (1) Health education, (2) Hospital news, (3) Medical consultation information, (4) Patient engagement, and (5) Official declaration. Health education posts are primarily associated with common health advice or misunderstood health facts. Hospital news is about any important event that is happening or has happened in the hospital, which serves as a self-promotion mechanism. Medical consultation information concerns special arrangements for outpatient visits, for example, those during holidays or temporary changes such as closures. Patient engagement collects public opinions about health service preferences, such as any suggestions in relation to hospital operations. Official declaration clarifies recent misinformed or misunderstood information about hospital administration or health staff. Of the 100 most commented posts, health education and hospital news had 44 posts and 33 posts, respectively. Table 3 presents more details on these.

Additionally, we examined the most commented posts on the 10 most popular accounts; 70% (7/10) of the most commented posts were hospital news, and a significant difference was also identified on their likes (min 0, max 7554, SD 2335.89), reposts (min 3, max 22,464, SD 7802.73), and comments (min 23, max 3428, SD 1245.36). Among them, three posts from the Children’s Hospital, Beijing Cancer Hospital, received more than 1000 comments. Results are presented in Table 4. Interestingly, a post related to hospital news on a general surgeon’s relief work in Kunming attracted nationwide attention because the public mistook the general surgeon for a measurement of the physician’s rank, rather than classification. Most of the comments were focused on why the hospital sent an ordinary doctor to the rescue and provided an explanation for the general surgeon. This post has now become a famous joke on the public’s poor knowledge about medical issues. We also found that almost all interactions happened among netizens.

**Table 2:** List of the top 10 popular social media accounts among the 705 best hospitals.

Sina Weibo account	No. of followers	No. of Tweets
Children’s Hospital, Capital Institute of Pediatrics	1,576,347	9631
Peking University, First Hospital	1,075,588	2511
Beijing Cancer Hospital	852,964	5178
Beijing Obstetrics and Gynecology Hospital, Capital Medical University	790,216	2132
Beijing Huilong Guan Hospital	650,954	1800
Beijing Children’s Hospitals, Capital Medical Hospital	648,248	4871
Tianjin Stomatological Hospital, Hospital of Stomatology, Nankai University	379,160	30,047
Children’s Hospital of Shanghai	268,430	3580
West China Hospital, Sichuan University	238,265	13,113
Peking University People’s Hospital	203,612	3,133

**Table 3:** Category of the 100 most commented posts.

Type of information	No. of posts	Example
Health education	44	Never shake your baby.
Hospital news	31	Peking University People’s Hospital has reached a new high! It is now first class, compared to Stanford University Hospital.
Medical consultation information	10	The hours of our outpatient visits are changing from 9-5 to 8-4, during the Spring Festival.
Patient engagement	8	Vote for your ideal type of psychiatrist.
Official declaration	5	An official declaration on the issue of workplace violence encountered by our nurses.

**Table 4:** Information about the most commented posts.

Posts	Type	No. of Likes	No. of Reposts	No. of Comments
A hard decision for the parents on their son’s liver replacement surgery because of the unbreakable cost (Children’s Hospital).	HN^a^	44	18	24
Our general surgeon Dr Jiang has flown to Kunming for disaster relief work (Peking University, First Hospital).	HN	1459	22464	2718
A new edition of *How far is cancer for you* has been released, and a brief introduction is provided (Beijing Cancer Hospital).	HE^b^	0	863	1222
For the convenience of the to-be-mothers, our hospital has implemented a 24×7 outpatient service scheme (Beijing Obstetrics and Gynecology Hospital).	HN	14	79	84
The reports on a patient visiting our psychiatry department, who was checked-in undressed, is not reliable (Beijing Huilong Guan Hospital).	OD^c^	0	53	42
A picture is more powerful than a thousand words (the hardworking doctors in Beijing Children’s Hospitals).	HN	880	1080	182
Women’s beauty relies on good sleep (Tianjin Stomatological Hospital).	HE	2	3	23
The music dream classroom, sponsored by a popular singer, in our hospital has opened (Children’s Hospital of Shanghai).	HN	1655	2917	736
Using a make-up blogger’s approach to promote polymerase chain reaction products by our medical students (West China Hospital, Sichuan University).	HN	7554	14573	3428
Our Prof Mu has participated in an international conference and has featured in a live broadcast medical plastic surgery section (Peking University People’s Hospital).	HN	55	329	63

^a^HN: hospital news.

^b^HE: health education.

^c^OD: official declaration.

## Discussion

### Hospital Social Media Adoption Varies Across Regions, With Regional Centralization Being Most Important

Hospitals in the Eastern region were the first to adopt social media, with the total number of hospitals adopting social media being larger than that of the other two regions. This may be attributable to the abundant resources available in the hospitals in the Eastern region [18,19], such as technicians and financial support. In addition, compared with the inland regions, they are more open-minded and responsive to changes in general [20,21]. From the perspective of diffusion, neighboring provinces are likely to present similar adoption patterns [22]; this is due to their proximity and exchange of information between hospital managers and staff being more likely. With this informal sharing, the number of hospitals that have appreciated the importance of social media in connecting with patients has increased, and social media has been identified as a common strategy to improve patient-physician communication. In addition, other factors may also play a critical role, such as the adoption of social media by public health agencies, the local popularity of social media platforms, and hospital manager characteristics (ie, gender, age, and openness toward social media).

### Transition of Usage From Communication to Service Orientation Makes WeChat More Attractive Among Hospitals

Sina Weibo initially attracted users because of its instant communication functionality. With the introduction of WeChat, Sina Weibo has gradually lost that advantage. As a new social media platform, WeChat offers greater functionality for engaging patients and offers nearly all the functions that Sina Weibo offers. More hospitals now turn to WeChat instead of Sina Weibo simply because WeChat reaches almost all ages, while users of Sina Weibo are decreasing and mostly comprise the younger age group. In addition, operating a microblogging account requires additional staff members to handle communication with internet citizens (netizens); this is time consuming and less rewarding. For example, a hospital’s Sina Weibo account is seen negatively as it distributes too much hospital information and offers less interaction with netizens. On the other hand, in the case of WeChat, individuals are more likely to review or provide a “thumbs up” or “like” and less likely to make comments because of its design. This makes operating a public WeChat account easier because it requires less attention compared with Sina Weibo. At the same time, the extra functionality that WeChat provides, such as appointment scheduling with the physicians and the checking of medical reports, makes it more attractive to users. Considering the long waiting times and difficulty in seeing expert physicians in China, WeChat has been very much welcomed by the public in China.

### Adoption of Social Media is Faster in Larger, Newer Hospitals With More Resources

During the early adoption of social media, Sina Weibo attracted newer and comprehensive hospitals. This may be attributed to the target group of Sina Weibo (primarily youth) and to the fact that recently built hospitals are more open-minded toward adopting new technology than the long established ones. Hospitals with a long history are seldom regarded as early social media users because more concerns may arise, for example, whether Sina Weibo is aligned with their traditional image. The best traditional hospitals have already established their reputation among patients. As an innovative method of engaging customers, social media application in hospitals is still unpredictable. Instead of enhancing communication abilities, they prefer to invest in patient safety, such as medical error reduction. In addition, comprehensive hospitals are larger in size than the specialized ones, and it is not surprising that they have more resources to support the use of Sina Weibo. Interestingly, specialized hospitals have more supporters than comprehensive ones [23]. A possible reason for this is that a specialized hospital can provide more tailored health information to its patients.

### Opening an Account is Never the End, and a Long Journey is Still Ahead for Hospitals

The verification of an official hospital account on a social media platform is only the first step. In fact, some of the studied hospitals discontinued their social media usage after only a few posts. This could be explained by the lack of staff, insufficient financial support, or simply little engagement from users. For hospitals, operating a social media account is not necessarily a task that they are judged on. Our observational study reveals that the content of hospital social media accounts is dominated by hospital news, and quite a few mutual communications between the public and the hospital are presented. Most of the interactions are generated by netizens, and the hospital seldom responds. Having official accounts means that hospitals need to interact with the public at all times. If a hospital fails to provide the public with the desired information, it may face intense criticism online. Therefore, operating social media accounts may put a hospital’s reputation in danger. Even though some pioneers have started to produce specific internal guidelines for hospitals’ social media usage [24,25] (eg, how to respond to difficult questions from the public, such as patient consultation for specific medical advice), most hospitals have no strict rules regarding its operation. For hospitals, it is definitely worth a second thought before creating official social media accounts. The process of monitoring, reviewing, and replying to patient concerns online may seem daunting to under-staffed information technology departments; however, it is crucial for developing a healthy online perception of the hospital and for enhancing relationships with current and prospective patients. In addition, although several hospitals have started realizing that patient comments posted online allow them to build and establish rapport and obtain feedback, rarely hospitals have successfully utilized them to further develop and improve their health care service delivery.

With the evolution of social media, it is quite possible that new forms of social media may replace once popular forms. Accordingly, another noteworthy problem—*A tale of two hospitals* on social media—arises for the hospitals that have both Sina Weibo and WeChat accounts. How do they operate their Sina Weibo and WeChat accounts—by simply synchronizing their content or by distinguishing it in some way?

### Limitations

This is a descriptive study of social media usage among the best tertiary hospitals in China. Our survey sample is limited to tertiary hospitals, with secondary or primary hospitals being excluded due to their extremely low social media adoption rates. Future studies must include a greater number of hospitals and compare variations in their social media adoption. Furthermore, our survey featured two predominant social media platforms, Sina Weibo and WeChat. Other social media platforms also deserve attention. For example, YouTube has been widely adopted by hospitals in the United States [26], while Youku (similar to YouTube) is seldom used by China’s hospitals [27], and it is worth investigating. In addition, this study is quantitative in nature; future studies, using a qualitative approach, should be conducted to explore specific strategies used by hospitals for social media adoption, such as who operates hospital accounts, what are the barriers and facilitators for them in engaging their patients on social media platforms [28], how hospital managers perceive the use of social media, and how does the social media account satisfy public needs [2].

### Conclusions

This study revealed the landscape of social media usage among China’s best tertiary hospitals. We found that social media adoption is prevalent among China’s best hospitals and that WeChat is more popular than Sina Weibo. Although the number of adopters across the three regions of China is increasing, early adopters are more cautious in terms of their social media strategies. In general, larger and newer hospitals with more resources are more likely to adopt social media. The transition of users from Sina Weibo to WeChat suggests that patients were not satisfied with mere communication functionality and they now place greater value on health services A long journey still lies ahead for hospitals in terms of operating their official social media accounts. However, if they actively monitor, manage, and engage with patients online, they should reap the benefits of building lasting relationships, enhancing satisfaction, and reassuring patients that their voices are being heard.

## Abbreviations

HE: health education
HN: hospital news
NHCPRC: National Health Commission of the People’s Republic of China
OD: official declaration
RQ: research questions
